# Supplementary material for: Spatiotemporal evolution characteristics of coordinated development of urbanization and ecological environment in eastern Russia—Perspectives from the 3D global trend and 2D plane analysis
Source: PLoS One. 2022 Jul 6;17(7):e0267272. doi: 10.1371/journal.pone.0267272 (PMC9258840; doi:10.1371/journal.pone.0267272)
Supplement: Attachment — Submitted filename: PONE-D-22-02247.docx [file pone.0267272.s001.docx]

Current paper studied the urbanization development level, eco-environment development level, and their coupling coordinated development degree during 2005-2018 in the eastern Russia from the perspectives of the 3D global trend and 2D plane analysis.

The content of the paper is good, but very technical and it is very difficult to follow what authors are trying to say

Overall significant work is needed on the language of the paper.

There are loose sentences and there are many sentences that start with “And”. Authors need to work on bringing them in proper language.

The content is good, but the style of writing is very technical and I feel it may not appeal a wider readership.

Also there are only limited references from past studies to compare and discussion section does not provide any comparison with earlier studies.

Currently introduction is lengthy, it will also help to add sub-headings in introduction section to make the reading easier and crispier

Better to keep labels for on all the maps as you have done for Fig 1

Certain terms such urbanization lagging need better wording and need to be defined in the paper properly

Discussion section does not provide the results of this study with any earlier study, need to add comparison and contrast for validation of the results

Conclusions are not properly formulated, need to work on the language to bring out exactly what are those 2-3 concluding points from this study

**Some of the comments are as follows:**

Line 13 – passive, instead of this paper studied-In this paper we studied

Line 55, 58, 78…., 653, 657, 662 – sentences starting with And

Line 72, 112 – research instead of researches

Line 75 – Shang analysed.. pls mention citation for Shang

Line 80 – mishchuk citation needed, pls check citation style for the journal

Line 89 – in the new era… need to specify approximate years
