# Supplementary material for: Spatiotemporal evolution characteristics of coordinated development of urbanization and ecological environment in eastern Russia—Perspectives from the 3D global trend and 2D plane analysis
Source: PLoS One. 2022 Jul 6;17(7):e0267272. doi: 10.1371/journal.pone.0267272 (PMC9258840; doi:10.1371/journal.pone.0267272)
Supplement: Attachment — Submitted filename: Response to reviewers .doc [file pone.0267272.s002.doc]

Thank you for your letter and for the reviewers’ comments concerning our manuscript. These comments are very helpful for revising and improving our paper. We have studied comments carefully and have made corrections which we hope meet with approval.

**Reviewer #1comments:**

This paper has a long introductory content, which needs to be further simplified. The introduction only points out the current national policy background and the situation of urbanization and ecological environment in eastern Russia, and there are too many background analyses.

**Response:** Thanks for your valuable comments and suggestions. We have simplified the introduction content. At the same time, we have deleted irrelevant policy documents and background analyses in the first and third paragraphs of the introduction, which makes the introduction more concise and logical.

The details are as follows:

**Introduction**

In 2013, China proposed “the Belt and Road” initiative, including “the Silk Road Economic Belt” initiative and “the 21st-Century Maritime Silk Road” initiative. In 2015, China issued the “Vision and Actions on Jointly Building Silk Road Economic Belt and 21st-Century Maritime Silk Road” initiative, which meant that “the Belt and Road” initiative came into a comprehensive promotion stage [1]. Among “the Belt and Road” initiative, “China- Mongolia- Russia economic corridor” initiative is constructed depending on the land transportation infrastructure [2]. Along China- Mongolia- Russia economic corridor, eastern Russia is an important neighbor area of Northeast China. As the key areas of “the Belt and Road” and “China- Mongolia- Russia economic corridor” initiatives, eastern Russia and Northeast China have came into the comprehensive opening-up and cooperation stage [2]. The coordinated development of urbanization and ecological environment is not only the global economic and social development trend, but also the important content of transnational economic cooperation between China and Russia.

Research on the urbanization and eco-environmental development in eastern Russia concentrates on the urbanization development process analysis, the urbanization development problems discussion, and the different eco-environmental problems research. (1) Urbanization development process analysis. Shang analyzed that the urbanization of eastern Russia had experienced three important stages [3], including the early eastward expansion of the Moscow Principality, the Russian expansion in the Amur River Basin, and the construction climax in the Tsarist Russian period. Relying on the rapid development of industrialization, the urbanization development of eastern Russia went through with military and administrative functions, which was from west to east developing, from the center to around radiating [4-6]. Mishchuk reviewed the urbanization immigrants characteristics in the Far East Federal District [7], including the European immigrants settling in the Far East Federal District, the forced and voluntary immigrants to the Far East Federal District, and the reverse migration flow formation in the Far East Federal District. (2) Urbanization development problems discussion. The urbanization development was rapidly promoted by the industrialization development in the Soviet Union. After the disintegration of the Soviet Union, Russia came into the transition process from the planned economy to the market economy. Russia’s urbanization was in the stagnation process because of the radical economic transformation and the regional serious crisis. The urbanization development was characterized by the shrinking and extinction of small cities, the negative growth of urban and rural population, the unstable development of urban system [8]. Gao and Li thought that the urban construction was developing slowly in the eastern Russia from 2010. There were a series of problems in the urbanization development, such as the slow urban population agglomeration, the small number of mega cities, the single industrial structure of small and medium-sized cities, the urban imperfect hierarchical scale structure, the uneven distribution of urban spatial structure [5, 9]. Kuleshov believed that the interaction between eastern Russia and Northeast China would solve the regional development imbalance and internal urbanization problems in eastern Russia [10]. (3) Different eco-environmental problems research. While developing mining, forest harvesting and high-consuming military enterprises, the eco-environmental problems such as the air pollution, water pollution, radioactive pollution, soil heavy metals, offshore pollution and forest resources loss became more and more serious in eastern Russia [11-12]. Bityukova believed that the urban air pollution severity mainly depended on the specialization degree of the industrial sectors in eastern Russia [13]. Vasilenko thought that the depletion and deterioration of water resources were mainly affected by human water resources activities in the southern areas of western Siberian Federal District [14]. Zhuravel analyzed the impact of coal mining on the ecological environment. He put forward the suggestions on sustainable and optimal operation efficiency in the southern Yakutia and Kuznetsk basins [15]. Based on the comprehensive index of human environmental intensity, Bityukova evaluated the urban eco-environment status, including the air and water pollution, solid waste quantity, thermal pollution and radiation pollution in eastern Russia. [16]. At present, research on the urbanization and eco-environment concentrates on unilateral analysis, there are relatively few studies on the coordinated development of urbanization and eco-environment in eastern Russia. Moreover, on the basis of long-time series of historical documents and historical data, Chinese scholars have carried out the qualitative research, judgment and discussion on urbanization and eco-environment in eastern Russia. However, due to the data limitations, the quantitative and visual research is obviously insufficient on the urbanization and eco-environment in eastern Russia. Especially in the past 10 years, research on the coordinated development of urbanization and eco-environment needs to be continuously updated in eastern Russia.

Eastern Russia contains twenty-one federal subjects. Among these federal subjects, the physical geography, energy resources, political affairs, economic level and social level are all different. After the disintegration of the Soviet Union, Eastern Russia has experienced severe economic crises. Its urbanization and eco-environment are still in the uncoordinated development condition. Eastern Russia is an important neighbor area of Northeast China. However, it is not clear for China to know the urbanization and eco-environment development conditions of eastern Russia. This phenomenon has seriously affected the cross-border cooperation in economy, trade, industry, transportation, communication, resources and environment between China and Russia. By studying the urbanization and eco-environment development patterns of eastern Russia, it would be helpful to select cross-border cooperation nodes, cooperation channels and cooperation platforms for China and Russia. It also would be helpful to explore the complementary growth points of regional cooperation, so as to form a coordinated development pattern for China and Russia. Therefore, it is very important to study the coordinated development pattern of urbanization and eco-environment in eastern Russia. In this paper, we studied the urbanization development level, eco-environment development level, and their coupling coordinated development degree during 2005-2018 in the eastern Russia from the perspectives of the 3D global trend and 2D plane analysis. First, combining with the Population-Economic-Sociology and Pressure-State-Response models, the urbanization index system and the eco-environment index system were constructed for eastern Russia. Second, based on the comprehensive weighting method of entropy weight method and variation coefficient weight method, the urbanization development level and eco-environment development level were calculated for eastern Russia. Third, the coupling coordinated development degree of the urbanization development level and eco-environment development level was measured by the coupling coordination model for eastern Russia. Finally, the spatial differentiation of the urbanization development level, the eco-environment development level, and their coupling coordinated development degree was performed respectively by the 3D global trend and 2D plane analysis in ArcGIS. In the theory, this research could enrich the theoretical systems of urban geography and urban ecology. It could provide guidance and reference for similar related research in other parts of the world, especially in China’s adjacent areas. In the practice, this research could clear the complementary points of bilateral cooperation for China and Russia. It could provide scientific reference for regional development planning, economic optimization layout, energy and resource development, infrastructure construction for the adjacent areas of China and Russia. It could provide suggestions for expanding the economic cooperation field and the economic investment scale for the border cities of China and Russia. It also could provide policy implications for the border trade, transportation facilities, border tourism, border cooperation zone, eco-environment protection of China and Russia.

Thanks!

**Reviewer #2comments:**

In terms of the index systems, the evaluation of urbanization and the evaluation of ecological environment are essentially two evaluations of this research. It is suggested to distinguish them in Table 1. Because this is not a set of evaluation index system, but two evaluation index systems.

**Response:** Thanks for your valuable comments and suggestions. We have distinguished the urbanization evaluation index system and eco-environment evaluation index system in Table 1 and Table 2 respectively. The details are as follows:

Table 1 Evaluation system of the urbanization in eastern Russia

| **First-Level**  **Indices** | **Second-Level**  **Indices** | **Third-Level**  **Indices**, **Unit** | **Attrib-ute** | **Entropy weight** | **Variation weight** | **Compre-hensive weight** |
| --- | --- | --- | --- | --- | --- | --- |
| Urbanization system | Population urbanization | Proportion of non-agricultural population, % | + | 0.01 | 0.02 | 0.01 |
|  |  | Population size (year-end), thousands | + | 0.09 | 0.09 | 0.09 |
|  |  | Population density, person/km^2^ | + | 0.16 | 0.13 | 0.15 |
|  |  | Annual average number of employees, thousands | + | 0.09 | 0.09 | 0.09 |
|  | Economic urbanization | Proportion of non-agricultural industrial output value in GDP, % | + | 0.00 | 0.01 | 0.003 |
|  |  | Regional GDP, million rubles | + | 0.12 | 0.11 | 0.12 |
|  |  | Integrated budget revenue, million rubles | + | 0.08 | 0.09 | 0.09 |
|  |  | Fixed capital investment (in real terms), million ruble | + | 0.12 | 0.11 | 0.12 |
|  |  | Total retail trade volume (current price), million rubles | + | 0.11 | 0.10 | 0.11 |
|  | Social urbanization | Highway operating mileage (at the end of the year), km | + | 0.10 | 0.10 | 0.10 |
|  |  | Number of hospital beds per 10000 population | + | 0.01 | 0.03 | 0.01 |
|  |  | Number of mobile radiotelephone connected user equipment per 1000 people, PCs | + | 0.02 | 0.03 | 0.02 |
|  |  | Number of students in general education institutions, thousands | + | 0.08 | 0.08 | 0.08 |
|  |  | Urban per capita residential housing area, m^2^ | + | 0.00 | 0.02 | 0.01 |

Table 2 Evaluation system of the eco-environment in eastern Russia

| **First-Level**  **Indices** | **Second-Level**  **Indices** | **Third-Level**  **Indices**, **Unit** | **Attrib-ute** | **Entropy weight** | **Variation weight** | **Compre-hensive weight** |
| --- | --- | --- | --- | --- | --- | --- |
| Ecological environment system | Ecological environment pressure | Pollutants discharged into the air from fixed sources, thousand tons | - | 0.11 | 0.11 | 0.11 |
|  |  | Polluted waste water discharged to surface water, million m^3^ | - | 0.09 | 0.09 | 0.09 |
|  |  | Area of forest fire, hm^2^ | - | 0.17 | 0.15 | 0.16 |
|  | Ecological environment state | Sown area of crops, thousand hm^2^ | + | 0.11 | 0.10 | 0.10 |
|  |  | Total grain harvest, thousand tons | + | 0.13 | 0.10 | 0.12 |
|  |  | Forest coverage, % | + | 0.01 | 0.02 | 0.01 |
|  |  | Area of forest land, thousand hm^2^ | + | 0.08 | 0.08 | 0.08 |
|  |  | Supply of fresh water, million m^3^ | + | 0.07 | 0.08 | 0.08 |
|  | Ecological environmental response | Share of captured and neutralized air pollutants in total waste pollutants in fixed pollution sources, % | + | 0.01 | 0.02 | 0.01 |
|  |  | Recycled water capacity, million m^3^ | + | 0.06 | 0.07 | 0.06 |
|  |  | Amount of pollutants collected from fixed sources, thousand tons | + | 0.10 | 0.10 | 0.10 |
|  |  | Reforestation area, thousand hm^2^ | + | 0.08 | 0.08 | 0.08 |

Thanks!

**Reviewer #3comments1:**

Overall significant work is needed on the language of the paper. There are loose sentences and there are many sentences that start with “And”. Authors need to work on bringing them in proper language.

**Response:** Thanks for your valuable comments and suggestions. We have looked for English experts in relevant research fields to modify and correct the whole paper in detail, so as to avoid the existence of loose sentences as far as possible and make the whole paper have more appropriate language. Please see the revised version of manuscript for details.

Thanks!

**Reviewer #3comments2:**

There are only limited references from past studies to compare and discussion section does not provide any comparison with earlier studies.

**Response:** Thanks for your valuable comments and suggestions. We have compared the previous research in the first paragraph of the discussion part, and then we have explained the differences and improvements between this paper and the previous research.

The details are as follows:

**Discussion**

Compared with earlier studies [3-16], first we studied the coordinated development of urbanization and ecological environment in eastern Russia, which could make up for the deficiency of a single study that only studied urbanization or ecological environment before. Second, we combined with the Population- Economic- Sociology and Pressure- State- Response models to construct urbanization evaluation system and eco-environment evaluation system, which could have more scientificity, logicality, focalization compared with previous urbanization or eco-environment studies in eastern Russia. Third, in the process of index weight determination, the combination of entropy weighting and coefficient variation weighting could not only avoid the uncertainty of the subjective weighting method, but also could get rid of the defects of the single objective weighting method. Finally, as for the previous historical qualitative research, the quantitative and visual research need to be continuously tracked and updated on the coordinated development of urbanization and ecological environment in eastern Russia.

In recent years, the urbanization and eco-environment are in the basic uncoordinated development stage in eastern Russia. In the future, on the basis of solving the contradiction between resource development and eco-environment protection, eastern Russia should promote the urbanization quality improvement. It should also coordinate the economic and social development strategies , so as to promote the economic balance development and the eco-environment protection among twenty-one federal subjects. On the basis of increasing regional financial support, optimizing regional infrastructure, protecting regional environment, developing innovative economic model, optimizing industrial structure, and encouraging governments functions, it should create conditions for economic, social and eco-environment development, driving the development of marginal areas with advantageous areas to realize diversified development.

Siberian Federal District should build a modern economic metropolitan area with Novosibirsk Region as the core, radiating Altay Territory, Kemerovo Region, Krasnoyarsk Territory and Irkutsk Region. It could integrate production factors, implement innovation driven development strategy, promote economic transformation in resource-based areas, and vigorously develop modern service industry. At the same time, it should develop the Arctic Development Zone on the basis of strengthening resource and energy exploration, restoring Arctic routes development, and protecting the original eco-environment. What’s more, it should develop the Northern Development Belt, focusing on automobile manufacturing, chemical industry, electric power and other industries. It should promote the Southern Development Belt, innovatively transforming its traditional economic sectors so as to develop industrial and economic cooperation zones with Asia Pacific and Central Asia.

Far East Federal District should stabilize the local population, attracting foreign labor force on the basis of the common development of eco-environment protection and economic interests. It should focus on planning infrastructure such as energy, transportation and information, especially paying attention to the cooperation with Northeast Asia. In the northeast marginal areas Far East Federal District, it should get rid of the inherent anti-immigration and ethnic discrimination, improving the stable resettlement system for labor immigrants. It also should formulate economic and urban construction investment plans, restoring its economic activity in order to attract immigrants from other regions. In the south of Far East Federal District, it should develop the agricultural industrial complexes and tourism leisure clusters in Khabarovsk Territory and Primorsky Territory. It should use eco-environment protection raw materials to transform the industrial structure, developing its diversified advantages of innovative resources. It also should take advantage of the ports’ advantages to enhance investment attraction, expanding production factors to other federal subject. In addition, it should deepen ecological and economic cooperation with northeast China, North Korea, South Korea and other neighboring countries, accelerating the planning and construction of transportation facilities synchronized with economic development.

Thanks once more!

**Reviewer #3comments3:**

Currently introduction is lengthy, it will also help to add sub-headings in introduction section to make the reading easier and crispier.

**Response:** Thanks for your valuable comments and suggestions. We have simplified the introduction content. At the same time, we have deleted irrelevant policy documents and background analyses in the first and third paragraphs of the introduction, which makes the introduction more concise and logical.

The details are as follows:

**Introduction**

In 2013, China proposed “the Belt and Road” initiative, including “the Silk Road Economic Belt” initiative and “the 21st-Century Maritime Silk Road” initiative. In 2015, China issued the “Vision and Actions on Jointly Building Silk Road Economic Belt and 21st-Century Maritime Silk Road” initiative, which meant that “the Belt and Road” initiative came into a comprehensive promotion stage [1]. Among “the Belt and Road” initiative, “China- Mongolia- Russia economic corridor” initiative is constructed depending on the land transportation infrastructure [2]. Along China- Mongolia- Russia economic corridor, eastern Russia is an important neighbor area of Northeast China. As the key areas of “the Belt and Road” and “China- Mongolia- Russia economic corridor” initiatives, eastern Russia and Northeast China have came into the comprehensive opening-up and cooperation stage [2]. The coordinated development of urbanization and ecological environment is not only the global economic and social development trend, but also the important content of transnational economic cooperation between China and Russia.

Research on the urbanization and eco-environmental development in eastern Russia concentrates on the urbanization development process analysis, the urbanization development problems discussion, and the different eco-environmental problems research. (1) Urbanization development process analysis. Shang analyzed that the urbanization of eastern Russia had experienced three important stages [3], including the early eastward expansion of the Moscow Principality, the Russian expansion in the Amur River Basin, and the construction climax in the Tsarist Russian period. Relying on the rapid development of industrialization, the urbanization development of eastern Russia went through with military and administrative functions, which was from west to east developing, from the center to around radiating [4-6]. Mishchuk reviewed the urbanization immigrants characteristics in the Far East Federal District [7], including the European immigrants settling in the Far East Federal District, the forced and voluntary immigrants to the Far East Federal District, and the reverse migration flow formation in the Far East Federal District. (2) Urbanization development problems discussion. The urbanization development was rapidly promoted by the industrialization development in the Soviet Union. After the disintegration of the Soviet Union, Russia came into the transition process from the planned economy to the market economy. Russia’s urbanization was in the stagnation process because of the radical economic transformation and the regional serious crisis. The urbanization development was characterized by the shrinking and extinction of small cities, the negative growth of urban and rural population, the unstable development of urban system [8]. Gao and Li thought that the urban construction was developing slowly in the eastern Russia from 2010. There were a series of problems in the urbanization development, such as the slow urban population agglomeration, the small number of mega cities, the single industrial structure of small and medium-sized cities, the urban imperfect hierarchical scale structure, the uneven distribution of urban spatial structure [5, 9]. Kuleshov believed that the interaction between eastern Russia and Northeast China would solve the regional development imbalance and internal urbanization problems in eastern Russia [10]. (3) Different eco-environmental problems research. While developing mining, forest harvesting and high-consuming military enterprises, the eco-environmental problems such as the air pollution, water pollution, radioactive pollution, soil heavy metals, offshore pollution and forest resources loss became more and more serious in eastern Russia [11-12]. Bityukova believed that the urban air pollution severity mainly depended on the specialization degree of the industrial sectors in eastern Russia [13]. Vasilenko thought that the depletion and deterioration of water resources were mainly affected by human water resources activities in the southern areas of western Siberian Federal District [14]. Zhuravel analyzed the impact of coal mining on the ecological environment. He put forward the suggestions on sustainable and optimal operation efficiency in the southern Yakutia and Kuznetsk basins [15]. Based on the comprehensive index of human environmental intensity, Bityukova evaluated the urban eco-environment status, including the air and water pollution, solid waste quantity, thermal pollution and radiation pollution in eastern Russia. [16]. At present, research on the urbanization and eco-environment concentrates on unilateral analysis, there are relatively few studies on the coordinated development of urbanization and eco-environment in eastern Russia. Moreover, on the basis of long-time series of historical documents and historical data, Chinese scholars have carried out the qualitative research, judgment and discussion on urbanization and eco-environment in eastern Russia. However, due to the data limitations, the quantitative and visual research is obviously insufficient on the urbanization and eco-environment in eastern Russia. Especially in the past 10 years, research on the coordinated development of urbanization and eco-environment needs to be continuously updated in eastern Russia.

Eastern Russia contains twenty-one federal subjects. Among these federal subjects, the physical geography, energy resources, political affairs, economic level and social level are all different. After the disintegration of the Soviet Union, Eastern Russia has experienced severe economic crises. Its urbanization and eco-environment are still in the uncoordinated development condition. Eastern Russia is an important neighbor area of Northeast China. However, it is not clear for China to know the urbanization and eco-environment development conditions of eastern Russia. This phenomenon has seriously affected the cross-border cooperation in economy, trade, industry, transportation, communication, resources and environment between China and Russia. By studying the urbanization and eco-environment development patterns of eastern Russia, it would be helpful to select cross-border cooperation nodes, cooperation channels and cooperation platforms for China and Russia. It also would be helpful to explore the complementary growth points of regional cooperation, so as to form a coordinated development pattern for China and Russia. Therefore, it is very important to study the coordinated development pattern of urbanization and eco-environment in eastern Russia. In this paper, we studied the urbanization development level, eco-environment development level, and their coupling coordinated development degree during 2005-2018 in the eastern Russia from the perspectives of the 3D global trend and 2D plane analysis. First, combining with the Population-Economic-Sociology and Pressure-State-Response models, the urbanization index system and the eco-environment index system were constructed for eastern Russia. Second, based on the comprehensive weighting method of entropy weight method and variation coefficient weight method, the urbanization development level and eco-environment development level were calculated for eastern Russia. Third, the coupling coordinated development degree of the urbanization development level and eco-environment development level was measured by the coupling coordination model for eastern Russia. Finally, the spatial differentiation of the urbanization development level, the eco-environment development level, and their coupling coordinated development degree was performed respectively by the 3D global trend and 2D plane analysis in ArcGIS. In the theory, this research could enrich the theoretical systems of urban geography and urban ecology. It could provide guidance and reference for similar related research in other parts of the world, especially in China’s adjacent areas. In the practice, this research could clear the complementary points of bilateral cooperation for China and Russia. It could provide scientific reference for regional development planning, economic optimization layout, energy and resource development, infrastructure construction for the adjacent areas of China and Russia. It could provide suggestions for expanding the economic cooperation field and the economic investment scale for the border cities of China and Russia. It also could provide policy implications for the border trade, transportation facilities, border tourism, border cooperation zone, eco-environment protection of China and Russia.

Thanks!

**Reviewer #3comments4:**

Better to keep labels for on all the maps as you have done for Fig 1.

**Response:** Thanks for your valuable comments and suggestions. We have kept labels for Fig 5 and Fig 7 as Fig 1.

The details are as follows:

a. Urbanization (2005)	b. Urbanization (2010)	c. Urbanization (2018)

d. Eco-environment (2005)	e. Eco-environment (2010)	f. Eco-environment (2018)

Note: a Omsk Region. b Tomsk Region. c Novosibirsk Region. d Altay Territory. e Kemerovo Region. f Republic of Khakasia. g Republic of Altay. h Republic of Tyva. i Krasnoyarsk Territory. j Irkutsk Region. k Republic of Buryatia. l Zabaikalsk Territory. m Republic of Sakha(Yakutia). n Chukotka Autonomous Area. o Kamchatka Territory. p Magadan Region. q Khabarovsk Territory. r Sakhalin Region. s Primorsky Territory. t Jewish Autonomous Area. u Amur Region.

This drawing has not been previously copyrighted. The authors created the image themselves.

**Fig 5. Spatial pattern changes of urbanization and eco-environment in eastern Russia from 2005 to 2018**

a. 2005	b. 2010	c. 2018

Note: a Omsk Region. b Tomsk Region. c Novosibirsk Region. d Altay Territory. e Kemerovo Region. f Republic of Khakasia. g Republic of Altay. h Republic of Tyva. i Krasnoyarsk Territory. j Irkutsk Region. k Republic of Buryatia. l Zabaikalsk Territory. m Republic of Sakha(Yakutia). n Chukotka Autonomous Area. o Kamchatka Territory. p Magadan Region. q Khabarovsk Territory. r Sakhalin Region. s Primorsky Territory. t Jewish Autonomous Area. u Amur Region.

This drawing has not been previously copyrighted. The authors created the image themselves.

**Fig 7. Spatial pattern changes of coupling coordination degree between urbanization and eco-environment in eastern Russia from 2005 to 2018**

Thanks!

**Reviewer #3comments5:**

Certain terms such urbanization lagging need better wording and need to be defined in the paper properly.

**Response:** Thanks for your valuable comments and suggestions. Referring to some relevant literatures, we have revised the “urbanization lagging” into “urbanization backwardness”. We have also explained the meaning of urbanization backwardness in the paper.

The details are as follows:

Table 3 Classification of coordinated development stages of urbanization and eco-environment

| **First-Level** | **Second-Level (Scope of *D*)** | **Third-Level(Comparison of *P*_1_ *and P*_2_)** | **Symbol** |
| --- | --- | --- | --- |
| Coordinated development | High-level coordinated development  (0.8<*D*≤1.0) | Eco-environment backwardness (*P*_1_-*P*_2_>0.1) | Ⅰ1 |
|  |  | Urbanization backwardness (*P*_2_-*P*_1_>0.1) | Ⅰ2 |
|  |  | Urbanization and eco-environment balance (0≤\|*P*_1_-*P*_2_\|≤0.1) | Ⅰ3 |
|  | Basic coordinated development  (0.5<*D*≤0.8) | Eco-environment backwardness (*P*_1_-*P*_2_>0.1) | Ⅱ1 |
|  |  | Urbanization backwardness (*P*_2_-*P*_1_>0.1) | Ⅱ2 |
|  |  | Urbanization and eco-environment balance (0≤\|*P*_1_-*P*_2_\|≤0.1) | Ⅱ3 |
| Uncoordinated development | Basic uncoordinated development  (0.3<*D*≤0.5) | Eco-environment backwardness (*P*_1_-*P*_2_>0.1) | Ⅲ1 |
|  |  | Urbanization backwardness (*P*_2_-*P*_1_>0.1) | Ⅲ2 |
|  |  | Urbanization and eco-environment balance (0≤\|*P*_1_-*P*_2_\|≤0.1) | Ⅲ3 |
|  | Serious uncoordinated development  (0<*D*≤0.3) | Eco-environment backwardness (*P*_1_-*P*_2_>0.1) | Ⅳ1 |
|  |  | Urbanization backwardness (*P*_2_-*P*_1_>0.1) | Ⅳ2 |
|  |  | Urbanization and eco-environment balance (0≤\|*P*_1_-*P*_2_\|≤0.1) | Ⅳ3 |

Note: Eco-environment backwardness means that the development level of eco-environment is lower than that of urbanization. Urbanization backwardness means that the development level of urbanization is lower than that of eco-environment.

Thanks!

**Reviewer #3comments6:**

Conclusions are not properly formulated, need to work on the language to bring out exactly what are those 2-3 concluding points from this study.

**Response:** Thanks for your valuable comments and suggestions. We have re-examined the full paper and tried to condense the results into 2-3 conclusions points exactly. We have also formulated the conclusions properly as much as possible.

The details are as follows:

**Conclusions**

From 2005 to 2018, the urbanization development level has shown the increasing trend, the eco-environment development level has shown a slight decreasing trend, and their coupling coordination development degree has shown an increasing trend in eastern Russia. The urbanization and eco-environment are in the basic uncoordinated development stage, which change from the urbanization backwardness to the urbanization and eco-environment balance in its internal system. The urbanization levels with population, economic and social aspects of the Siberian Federal District are all higher than those of the Far East Federal District. The eco-environment levels with pressure, state and response aspects of the Siberian Federal District are all balanced to those of the Far East Federal District. The urbanization and ecological environment of the Siberian Federal District and Far East Federal District are in the basic uncoordinated development stage. Among them, Krasnoyarsk Territory, Novosibirsk Region, Kemerovo Region, Altay Territory and Irkutsk Region have high coupling coordination development degree, other federal subjects are still in the uncoordinated development stage.

Spatially, from the 3D perspective, the urbanization development level, the eco-environment development level and their coupling coordination development degree all show the spatial characteristics of “High West, Low East” and “High Center, Low South, Low North” in eastern Russia. From the 2D perspective, the urbanization development level, the eco-environment development level and their coupling coordination development degree characterize the “High West, Low East” pattern in eastern Russia. The federal subjects with high urbanization level, eco-environment level and their coupling coordination development degree are mainly distributed in the serious areas of Novosibirsk Region - Altay Territory- Kemerovo Region- Krasnoyarsk Territory- Irkutsk Region. The federal subjects with low ones are mainly located in the Republic of Altay in the south of Siberian Federal District, and Chukotka Autonomous Area in the northeast of Far East Federal District.

From 2005 to 2018, Novosibirsk Region, Altay Territory, Kemerovo Region, Krasnoyarsk Territory and Irkutsk Region change from the basic uncoordinated development type into the basic coordinated development type, which their internal system changes from the urbanization and eco-environment balance into the eco-environment backwardness. Primorsky Territory, Khabarovsk Territory, Republic of Buryatia, Amur Region, Sakhalin Region, Republic of Sakha(Yakutia) and Omsk Region are still in the basic uncoordinated development type, which their internal system changes from the urbanization backwardness to the urbanization and eco-environment balance. Zabaikalsk Territory, Tomsk Region and Republic of Khakasia are still in the basic uncoordinated development type, Republic of Tyva, Kamchatka Territory and Magadan Region change from the serious uncoordinated development type into the basic uncoordinated development type, Republic of Altay, Jewish Autonomous Area and Chukotka Autonomous Area are still in the serious uncoordinated development type, which their internal system is still in the urbanization backwardness type.

Thanks!

**Reviewer #3comments7:**

Line 13 – passive, instead of this paper studied-In this paper we studied

Line 55, 58, 78…., 653, 657, 662 – sentences starting with And

Line 72, 112 – research instead of researches

Line 75 – Shang analysed.. pls mention citation for Shang

Line 80 – mishchuk citation needed, pls check citation style for the journal

Line 89 – in the new era… need to specify approximate years

Line 13 – passive, instead of this paper studied-In this paper we studied

Line 55, 58, 78…., 653, 657, 662 – sentences starting with And

Line 72, 112 – research instead of researches

Line 75 – Shang analysed.. pls mention citation for Shang

Line 80 – mishchuk citation needed, pls check citation style for the journal

Line 89 – in the new era… need to specify approximate years

**Response:** Thanks for your valuable comments and suggestions. Through the inspection and correction of the paper, the detailed problems raised by experts have been modified. “this paper studied” has been modified to “In this paper, we studied”. Sentences starting with “And” has been corrected. “researches” has been instead of “research”. “in the new era” has been specified as “From 2010”. “Shang analysed” has been mentioned citation in the Reference 3. We have checked citation style of “Mishchuk citation” in the Reference 7. Please see the revised version of manuscript for details.

Thanks once more!
